# Development and Optimization of Label-Free Quantitative Proteomics under Different Crossing Periods of Bottle Gourd

**DOI:** 10.3390/cimb45020088

**Published:** 2023-02-06

**Authors:** Anurag Malik, Virender Singh Mor, Himani Punia, D. S. Duhan, Jayanti Tokas, Axay Bhuker, Mohammed Nasser Alyemeni, Awais Shakoor

**Affiliations:** 1 Department of Seed Science & Technology, College of Agriculture, CCS Haryana Agricultural University, Hisar 125 004, India; 2 Department of Biochemistry, College of Basic Sciences & Humanities, CCS Haryana Agricultural University, Hisar 125 004, India; 3 Department of Vegetable Science, College of Agriculture, CCS Haryana Agricultural University, Hisar 125 004, India; 4 Botany and Microbiology Department, College of Science, King Saud University, Riyadh 11451, Saudi Arabia; 5 Department of Environment and Soil Sciences, University of Lleida, 25198 Lleida, Spain

**Keywords:** bottle gourd, crossing periods, label-free quantitation, plant proteomics, seed vigor, SDS-PAGE

## Abstract

Bottle gourd, a common vegetable in the human diet, has been valued for its medicinal and energetic properties. In this experiment, the time-resolved analysis of the changes in the proteins’ electrophoretic patterning of the seed development at different crossing periods was studied in bottle gourd using label-free quantitative proteomics. Hybrid HBGH-35 had the highest observed protein levels at the 4th week of the crossing period (F_4_) compared to the parental lines, viz. G-2 (M) and Pusa Naveen (F). The crossing period is significantly correlated with grain filling and reserve accumulation. The observed protein expression profile after storage was related to seed maturation and grain filling in bottle gourds. A total of 2517 proteins were identified in differentially treated bottle gourd fruits, and 372 proteins were differentially expressed between different crossing periods. Proteins related to carbohydrate and energy metabolism, anthocyanin biosynthesis, cell stress response, and fruit firmness were characterized and quantified. Some proteins were involved in the development, while others were engaged in desiccation and the early grain-filling stage. F4 was distinguished by an increase in the accumulation of low molecular weight proteins and enzymes such as amylase, a serine protease, and trypsin inhibitors. The seed vigor also followed similar patterns of differential expression of seed storage proteins. Our findings defined a new window during seed production, which showed that at F_4_, maximum photosynthetic assimilates accumulated, resulting in an enhanced source–sink relationship and improved seed production. Our study attempts to observe the protein expression profiling pattern under different crossing periods using label-free quantitative proteomics in bottle gourd. It will facilitate future detailed investigation of the protein associated with quality traits and the agronomic importance of bottle gourd through selective breeding programs.

## 1. Introduction

Bottle gourd (*Lagenaria siceraria* (Molina) Standl) is an edible, useful, and medicinal vegetable crop that belongs to the Cucurbitaceae family. It is a cross-pollinated vegetable, and the amount of pollination ranges from 94% to 99%. The degree of cross-pollination also depends on several factors: flowering time, temperature, wind velocity and direction, planting design, insect population, and genotypic nature, ultimately determining the kernel quality [1]. In the cucurbit’s vegetable crop, signs of the reproductive phase emerge approximately six to seven weeks after the planting when the flowering starts. Seed quality is a significant aspect of agricultural production and food security, especially during the growing uncertainty caused by climate change and abiotic factors [2]. It has a significant impact on output and resource efficiency when evaluating crop sustainability [3]. Hybrids hold the plant’s whole genetic combination, making them a distribution platform for agricultural biotechnology and crop enhancement [4]. Manufacturers demand high-quality seeds to maximize their profits for crop cultivation, so their benefits would not be compromised during their field trials. Farmers constantly need high-quality sources to ensure efficient and effective plantations; thus, companies must sell high-quality seeds to maintain their competitive positions in markets [5].

The practical and inexpensive processing of seeds is an essential prerequisite for the successful hybrid production of seeds [6]. The efficiency of hybrid seed production depends on multiple factors, such as selecting an appropriate agro-climate location, a suitable season, improved floral synchronization for enhanced seed setting through the right crossing period and supplementary pollination techniques, etc. [1]. Owing to the agronomical, economic, and environmental significance and understanding of the molecular and genetic dynamics governing seed development processes, recent genome-wide studies have focused on programs considering quality and yield [5,7]. Another important agronomical trait is seed size, which directly influences grain yield and is determined by storage reserves at physiological maturity [8]. 

Higher value-added seeds could be produced by knowing the seed storage proteins, which account for 40–70% of storage proteins, enzymes, housekeeping proteins, and other proteins [9]. Quality is an essential aspect of crop production, and it demands specific quality and functionality during development [10]. During germination, enzymatic hydrolysis of storage proteins occurs, where proteases convert them into soluble peptides to produce free amino acids that are transported to the growing embryonic axis to maintain growth and a source of energy [11]. Several complex and interrelated processes are involved in seed development, which is highly malleable [8]. The development of the embryo is accompanied by desiccation tolerance, the final phase of seed maturation [12]. Under adverse conditions, the quality of the seed deteriorates [13]. Seed deterioration is a biological process determined by a range of physical, biochemical, and physiological changes that commence at physiological maturity and subsequently progress, which negatively affect metabolic activities, loss of seed viability, reduced seed quality, and culminate in seed death [14]. Therefore, seed vigor could be described as the energy level of viable seeds in sustainable agriculture, and the complicated interplay determines the interaction between genetic and environmental factors. Therefore, the justifications for the variability in this performance are cumbersome and remain unexplained.

Label-free proteomics is a method based on the spectral count of mass peaks and is convenient and reliable in proteomics studies [15,16]. Improvements in normalization methods have greatly improved the accuracy of label-free proteomics that rely on mass spectrometry stability [17,18,19]. Moreover, the unlabeled method reduces experimental error and is more accurate compared with the labeling method, and label-free quantitative proteomic approaches recognize the deepest proteome coverage, which is advantageous for the initial exploration of interesting proteins produced in bottle gourd during different crossing periods [20,21,22].

The proteomics study consolidates the information needed to visualize, discover, and compare the proteins and mechanisms linked with crop physiology and has also become the most commonly used approach for identifying proteins present in fruits and vegetables’ biological systems [23,24]. Analysis of electrophoretic patterns via proteomics better indicates cellular and metabolic activities in bottle gourd [2,7,25,26,27]. Very little information is available on the protein extraction of vegetables, chiefly bottle gourd. To our knowledge, this is the first time-resolved study at the proteome level under different crossing periods for seed production in bottle gourd. Quality seed plays an essential role in enhancing agricultural productivity as well as the national economy. To achieve agricultural development goals, the supply of viable and vigorous seeds during planting time is crucial, as a healthy seed is a prerequisite for exploiting additional inputs’ full potential. Therefore, the present study aims to understand and elucidate the proteome and transcriptome functional interactions, particularly those with unique features, evaluate the best crossing period for quality seed development, and subsequently regulate the deposition of storage reserves during seed maturation.

## 2. Materials and Methods

### 2.1. Plant Materials and Experimental Design

Seeds of the bottle gourd parental lines, G-2 Line (male) and Pusa Naveen (female), were procured from the Department of Vegetable Science to produce Hybrid HBGH-35. The experiment was conducted at Seed Science and Technology (29.1416° N, 75.7112° E, with an average elevation of 215 m (705 ft) above mean sea level) during the Kharif season of 2017–2018. The soil type of the experimental field is sandy loam. The concentrations of organic matter percent, total nitrogen, available phosphate, and rapidly available phosphate in the uppermost 20–30 cm of soil were 0.49%, 182 kg/ha, 18 kg/ha, and 285 kg/ha, respectively. The climate is semi-arid, with freezing winters and hot, dry, desiccating winds during the summer. All the laboratory analyses were performed at the Seed Biotechnology Laboratory and the Centre of Bio-nanotechnology (Central Laboratory) of CCS Haryana Agricultural University, Hisar, India. 

### 2.2. Experimental Manipulations

The seed of parental lines was sown in the field during 2019–2020 and 2020–2021. Being an indeterminate crop, flowering in bottle gourds continues for around two months. Sowing took place on 17 July, and flowering began in both parents in the first week of September. The crossing period is divided into five meteorological weeks: F1 (4–10 September), F2 (11–17 September), F3 (18–24 September), F4 (25 September to 1 October), and F5 (2–8 October). The emasculation and pollination work were performed in both parental lines with the help of needles, scalpers, and forceps. The pollen was collected from male flowers and dusted on the stigmatic surface of emasculated female flowers for hybrid seed production. Manual emasculation and dusting were continued throughout the crossing period. Subsequently, all the floral buds emerging beyond 15 days after the crossing period were pinched off to ensure better growth and production of crossed fruits. At the start of the crossing period in hybrid and parental line seed production, the male flower was chosen in the parental line and bagged with paper bags the previous evening, pinched off the next morning, and carefully kept in a glass container.

The leaves and seeds were harvested at different crossing periods: fruit set during the first week of the crossing period (F1), fruit set during the second week of the crossing period (F2), fruit set during the third week of the crossing period (F3), fruit set during the fourth week of the crossing period (F4), and fruit set during the fifth week of the crossing period (F5), and kept for hybrid and parental line seed production. Tender leaves present at the axillary buds were chosen for this study. The weather data during different agro-meteorological crossing weeks are shown in Table 1 and Table 2. The leaves were excised from the vines (growing points) from each crossing period and immediately flash-frozen in liquid nitrogen and stored at −80 °C. Every experiment was carried out in three biological replicates and five technical triplicates. For protein analysis, seeds were manually extracted from each crossing period. After extraction, seeds were dried in the shade to lower the moisture content to 8% and were frozen in liquid nitrogen, and stored in sealed containers at −80 °C.

### 2.3. Seed Vigor

Seedling vigor indexes I and II were calculated as per the formula described [28]. 

Seedling vigor index-I = germination (%) × seedling length (cm).

Seedling vigor index-II = germination (%) × seedling dry weight (mg).

### 2.4. Optimization of the Methodology with Protein Extraction Methods

#### 2.4.1. Extraction from the Lysis Buffer

The freshly collected bottle gourd leaves under different crossing periods (F1, F2, F3, and F4) were grounded in liquid nitrogen (−196 °C) by using a pre-chilled pestle and mortar for the extraction of proteins. To this fine powder, lysis buffer (2% CHAPS, 30 mM Tris 2 M, thiourea, and 7 M urea) was added for precipitation, and sonication was performed in a sonicator (UP200S, Hielscher) for about 10 s for up to four cycles. The precipitated solution was kept on a rotator for 2 h for mixing, and after that, it was centrifuged at 15,000× *g* for 20 min. The supernatant obtained was stored at −80 °C for further use.

#### 2.4.2. Sucrose Extraction 

Proteins were extracted using sucrose [29], with some modifications. Using a homogenizer, 5 g of bottle gourd leaf and seed were homogenized in liquid nitrogen using 20 mL of extraction buffer (50 mM sodium borate, 50 mM ascorbic acid, 1% mercaptoethanol, and 1 mM PMSF) at 4 °C for 1 min (IKAT-18, Staufen, Germany). Following that, the material was centrifuged at 35,000× *g* for 30 min at 4 °C (Thermo Scientific, Loughborough, UK). Then, 50 mL of cold 0.1 M ammonium acetate in methanol was added to the clear supernatant. Protein precipitation was achieved by incubating the samples overnight at −20 °C.

Using a homogenizer (IKAT 18, Staufen, Germany), 5 g of bottle gourd leaf and seed were homogenized in liquid nitrogen with 20 mL of extraction buffer (50 mM sodium borate, 50 mM ascorbic acid, 1% mercaptoethanol, and 1 mM PMSF) at 4 °C for 1 min. The sample was then centrifuged (Thermo Scientific, Loughborough, UK) at 35,000× *g* for 30 min at 4 °C. To this clear supernatant, 50 mL of cold 0.1 M ammonium acetate in methanol was added. The samples were incubated overnight at −20 °C for protein precipitation. 

### 2.5. TCA Extraction Methods

#### 2.5.1. 10% TCA with 0.07% β-ME and 1 mM PMSF

Fresh leaves and seeds were homogenized in liquid nitrogen and precipitated in 10% trichloroacetic acid (TCA) diluted in cold acetone containing 0.07% mercaptoethanol and 1 mM of phenylmethylsulfonyl fluoride. The suspension was centrifuged at 15,000× *g* for 30 min at 4 °C, and the pellet was air-dried.

#### 2.5.2. 10% TCA with 0.07% β-ME

The seed and leaf samples were homogenized in liquid nitrogen. After crushing, 10% trichloroacetic acid dissolved in cold acetone containing 0.07% mercaptoethanol was added. The suspension was sonicated for 1 min with a 10 s interval and kept at −20 °C for 1 h. It was centrifuged at 20,000× *g* for 20 min at 4 °C, and the protein pellet was washed.

#### 2.5.3. Acetone-Phenol Extraction

The tissue was re-suspended in 10 mL of cold acetone, vortexed for 5 min, and centrifuged at 10,000× *g* for 5 min at 4 °C. The polyphenol-free sample was then air-dried before protein extraction, and this procedure was repeated three times. Then, 1 g of tissue was mixed with 2.5 mL of chilled Tris-buffered phenol, pH 8.8 (Fisher Scientific, Loughborough, UK), and 1 mL of chilled extraction buffer (0.2 M Tris-base pH 8.5, 10 mM EDTA, 0.4% mercaptoethanol, and 1 M sucrose), then vortexed on ice for 30 min and centrifuged for 10 min at 5000× *g*. The aqueous phase was again vortexed for 1–2 min after adding extraction buffer and Tris-buffered phenol. The material was then centrifuged for 10 min at 4 °C at 5000× *g*. The phenol phase was mixed with the initial phenol extraction phase before being centrifuged for 5 min at 10,000× *g*. The clear aqueous phase was decanted into a new tube. The phenolic phase was vortexed well after adding 25 mL of cool 0.1 M ammonium acetate in cold methanol. The material was then incubated overnight at −20 °C. The precipitate was collected by centrifugation at 20,000× *g* for 20 min at 4 °C. 

#### 2.5.4. Sodium Dodecyl Sulfate (SDS) Extraction

In this method, proteins were extracted using SDS according to the procedure proposed by Toledo et al. [29], with slight modifications. Here, 500 μL of extraction buffer containing 0.0625 M Tris-HCl, pH 6.25, 2% sodium dodecyl sulfate (SDS), 10% glycerol, 5% ME, and 0.001% bromophenol blue was used to extract the sample. The samples were vortexed and left at room temperature for an overnight period. Then, 4% CHAPS was added into the protein sample and then boiled for 5 min, and the precipitate was collected by centrifugation at 18,000× *g* for 15 min at 15 °C.

### 2.6. Precipitation Methods

As was stated above, the soluble proteins in the supernatant were precipitated using different methods.

#### 2.6.1. Precipitation with Acetone Containing β-ME and PMSF

The protein pellet was washed three times with cold acetone containing 0.07% mercaptoethanol and 1 mM of PMSF, then centrifuged at 10,000× *g* for 30 min and incubated at −20 °C for 1 h. Again, the pellet was centrifuged at 25,000× *g* for 25 min and then dissolved in lysis buffer. 

#### 2.6.2. Precipitation with Acetone Containing β-ME 

The protein pellet was washed twice in acetone containing 0.07% ME and 0.07% (*w*/*v*) DTT for 15 min at 20,000× *g*. The mixture was stored at −20 °C for 1 h and centrifuged at 20,000× *g* for 25 min. Finally, the pellet obtained was resuspended in a lysis buffer for further use.

#### 2.6.3. Protein Precipitation with Ammonium Acetate

The protein pellet was washed once with 0.1 M ammonium acetate in cold methanol, twice with cold 80% acetone, and once with cold 70% methanol. The final resulting pellet was air-dried.

#### 2.6.4. Clean-Up

The supernatant containing soluble protein samples was precipitated, followed by their clean-up. All the interfering compounds, such as detergents, lipids, nucleic acids, and other salts, were removed using a 2D-Clean-up kit (Bio-Rad Laboratories, Hercules, CA, USA). To the supernatant, precipitation agents were added for precipitation, and it was then centrifuged at 15,000× *g* for 5 min. The pellet was again washed with washing reagents (1 and 2), followed by centrifugation at 12,000× *g* for 10 min. The clear pellet was incubated for 1 h at −20 °C. After the incubation, it was again centrifuged. The pellet was air-dried at room temperature and resuspended in lysis buffer with gentle shaking (to avoid bubbling). 

### 2.7. Quantification of Proteins

The total soluble protein in the re-suspended pellet was quantified by a 2D Quant kit (Thermo Fisher Scientific Inc., Waltham, MA, USA) per the manufacturer’s instructions and measured in an ELISA Plate Reader (M200 pro-NanoQuant, TECAN) using bovine serum albumin (BSA) as a standard.

### 2.8. Electrophoresis 

The proteins present in the leaf and seed extracts were analyzed by sodium dodecyl sulfate-polyacrylamide gel electrophoresis (SDS-PAGE). The stacking gel (4%) and resolving gel (12%) were used to separate the proteins in the MiniVE gel electrophoresis apparatus (Bio-Rad Laboratories, Hercules, CA, USA). Then, 30 µg of sample, as quantified by the BSA standard, was loaded in each well. Coomassie Brilliant Blue (CBB) G 250 (Bio-Rad Laboratories, Hercules, CA, USA) was used to stain the gels, followed by dissolving and de-staining in acetic acid and methanol. The Gel Pro Analyzer version 3.3 was used to scan the gel slab. The standard molecular weight marker determined the polypeptides’ molecular weight (Thermo Fisher Scientific Inc., Waltham, MA, USA).

### 2.9. In-Gel Digestion of the SDS-PAGE Separated Proteins

For in-gel digestion, 30 μg of protein was loaded into each well, excised using a scalpel as indicated, and then added to Eppendorf tubes (1.5 mL). Excised pieces were subjected to de-staining using 50 mM of DTT, diluted in 50 mM of ABC, and incubated at 56 °C for 2 h. After the reduction step, the DTT solution was removed and 100 mM of IAA in 50 mM of ABC was added for the alkylation of cysteine residues. Then, the IAA solution was removed from the gel pieces and washed three times with 50 mM of ABC. The gel pieces were then air-dried three times for 5 min at room temperature. Following this, 50 mM of ABC buffer, containing 12.5 ng/μL of sequencing-grade modified trypsin (freshly prepared), was added. Then, 40 mM of ABC was added to cover the gel pieces and incubated overnight at 37 °C.

### 2.10. Peptide Extraction

After trypsin digestion, 10% *v*/*v* formic acid and the supernatant were incubated for 10 min at 37 °C. Peptides were extracted from the digested pieces by sonicating for 10 min with ACN/H_2_O (50:50, *v*/*v*; 50 µL) + 0.1% *v*/*v* TFA. The pooled supernatant was dried by spinning tubes in a speed vac and stored at −80 °C. For use in mass pac, the lyophilized sample was reconstituted with 1% trifluoroacetic acid (TFA) (after this step, desalting was performed).

### 2.11. Peptide Desalting 

The dried samples were reconstituted with 0.1% formic acid. Zip-tip C18 (Millipore Corporation, Bedford, MA, USA) was activated with 100% ACN. The samples were loaded onto a zip-tip, and the peptide was bound to C18 material. A zip-tip was loaded with 20 µL of 0.1% formic acid and 60% ACN, and the elute was collected in a new Eppendorf tube and stored at −80 °C until LC-MS/MS quantification.

### 2.12. Nano LC-MS/MS

The samples were then analyzed using an LC system (EASY-nLC 1200; Thermo Fisher Scientific) coupled to an MS (Orbitrap Fusion ETD MS; Thermo Fisher Scientific), equipped with a LC pre-column (75 μm × 2 cm, Nanoviper C18, 3 μm) at a flow rate of 300 nL/min. The peptide ions were detected using the MS (Orbitrap LC-MS, Thermo Fisher Scientific) with the installed Xcalibur software (version 2.0.7; Thermo Fisher Scientific). The MS was used to acquire full-scan mass spectra ranging from 375 to 1800 *m*/*z* with a resolution of 12,000. The acquired MS spectra were used for protein identification.

### 2.13. Differential Analysis of MS Data

Label-free quantification was also performed with Proteome Discoverer (PD) 2.2 (Version 2.2.0.388; Thermo Scientific), and the differential analysis of the relative abundance of proteins between samples was calculated. To determine the functional role of the proteins identified in the MS analysis, functional categorization of identified proteins was performed using Gene Ontology (GO) software, PANTHER (Protein Analysis Through Evolutionary Relationships). Pathway mapping of identified proteins was performed using the Kyoto Encyclopedia of Genes and Genomes (KEGG) databases.

### 2.14. Data Analysis

The LFQ intensities derived from all the evaluated PD samples were considered for statistical analysis. Each distinctive band was visually labeled with a number. The presence and absence were scored as 1 or 0, respectively. The data were statistically analyzed by the Gel Doc 2000 Bio-Rad system.

## 3. Results 

### 3.1. Agro-Metrological Conditions and Productivity

Currently, bottle gourd is considered neglected and is an underutilized species globally. It has immense potential, but its possible widespread adoption is restricted due to a lack of evidence regarding its morphology, physiology, and nutritional benefits. The adaptation and distribution of bottle gourd are bi-hemispherical, and therefore, it grows well in both tropical and temperate regions. The weather data during different agro-meteorological crossing weeks are shown in Table 1 and Table 2. During the Kharif (summer) season, the optimum temperature favoring the seed set at the F4 crossing period was 35 °C (Table 2). In contrast, relative humidity, bright sunshine hours (BRI), evaporation rate, and maximum precipitation rate were around 87%, 6.8, 4.2, and 1.9 mm, respectively, favoring the maximum seed production with full vigor potential (Table 1). 

### 3.2. Seed Vigor Analysis

**Figure 1:**
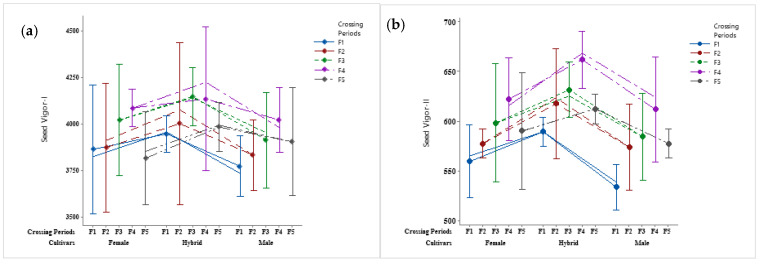
The seed vigor potential of bottle gourd hybrid HBGH-35 and two parental lines, female Pusa Naveen and male G2-line. (**a**) Seed vigor I and (**b**) seed vigor II.

**Figure 2:**
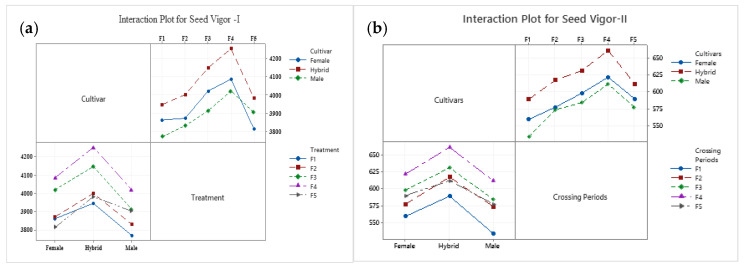
As an interaction plot, the seed vigor potential of bottle gourd hybrid HBGH-35 and two parental lines, female Pusa Naveen and male G2-line, were plotted. (**a**) Seed vigor I and (**b**) seed vigor II.

The results revealed that at the fourth week of the crossing period (F4), the accumulation of seed reserves was the maximum in Hybrid HBGH-35, which thus had full seed vigor potential, followed by the female parental line (Pusa Naveen), while the minimum was in the male parental line (G2-line) (Figure 1a,b). The temperature plays a crucial role in seed setting and fruit development (Table 1 and Table 2). At a temperature of 34 °C and a relative humidity of 87%, the F4 crossing period had maximum seed potential. The interaction plot between the bottle gourd parental lines and the hybrid revealed that both the hybrid HBGH-35 and F4 crossing periods had the highest seed vigor I and II (Figure 2a,b). 

### 3.3. Optimization of the Methodology 

**Figure 3:**
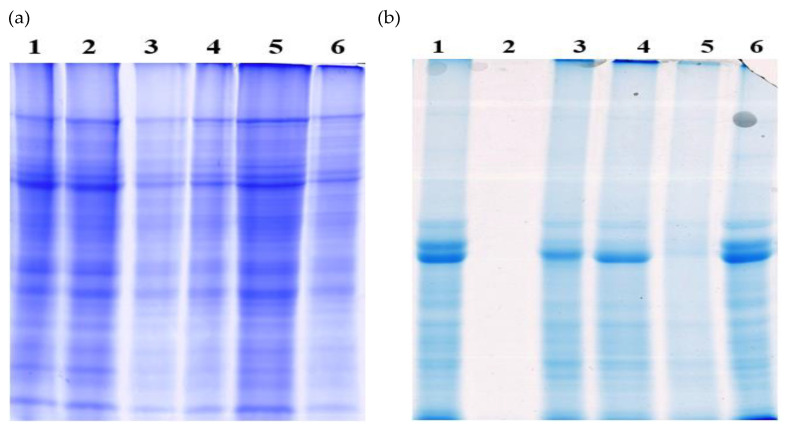

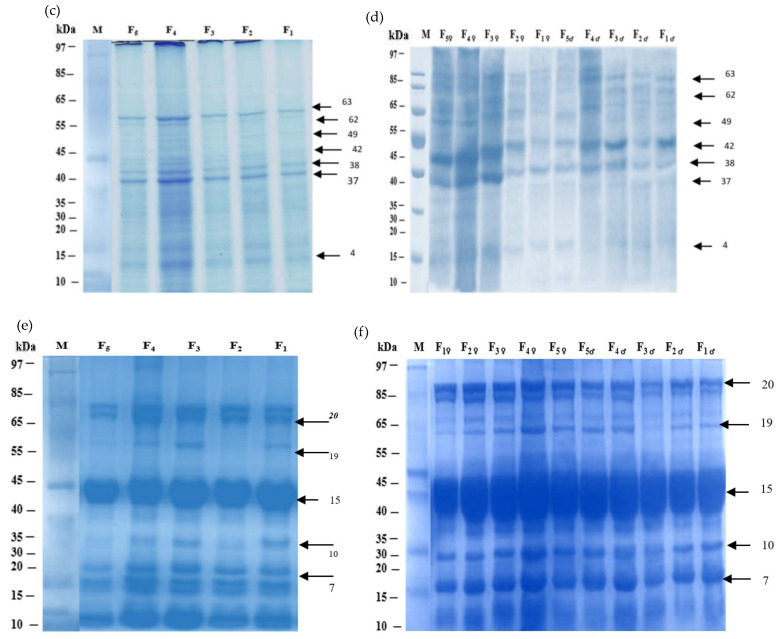
(**a**) Gel showing different protein extraction methods in bottle gourd leaves. Lane 1, SDS; Lane 2, acetone-phenol; Lane 3, 10% TCA containing β-ME; Lane 4, TCA with β-ME and 1 mM PMSF; Lane 5, sucrose; Lane 6: lysis buffer. (**b**) Lane 1, lysis buffer; Lane 2, sucrose; Lane 3, 10% TCA containing β-ME and 1 mM PMSF; Lane 4, TCA with β-ME; Lane 5, acetone-phenol; Lane 6, SDS. (**c**) SDS-PAGE of extractable proteins in bottle gourd leaves. Hybrid HBGH-35: Lane 1, F1; Lane 2, F2; Lane 3, F3; Lane 4, F4; Lane 5, F5. (**d**) Lane 1, F1 in male; Lane 2, F2 in male; Lane 3, F3 in male; Lane 4, F4 in male; Lane 5, F5 in male; Lane 6, F1 in the female; Lane 7, F2 in the female; Lane 8, F3 in the female; Lane 9, F4 in the female; Lane 10, F5 in the female. (**e**) SDS-PAGE analysis of extractable proteins in bottle gourd seeds. Hybrid HBGH-35: Lane 1, F1; Lane 2, F2; Lane 3, F3; Lane 4, F4; Lane 5, F5. (**f**) Lane 1, F1 male; Lane 2, F2 male; Lane 3, F3 male; Lane 4, F4 male; Lane 5, F5 male; Lane 6, F1 female; Lane 7, F2 female; Lane 8, F3 female; Lane 9, F4 female; Lane 10, F5 female; M: marker; kDa: kilodaltons (molecular weight standards).

Preliminary research was conducted to establish the appropriate method to extract protein from bottle gourd. The protein extracted from the SDS reagent (Figure 3a, lane 1) and TCA containing ME, as well as TCA containing mercaptoethanol and 1 mM of PMSF, did not yield reproducible results (Figure 3a, lanes 3 and 4). The lysis buffer protein extraction method exhibited substantial products with sharp band intensities and band strengths, followed by their clean-up (Figure 3a, lane 6). The protein extracted with sucrose (Figure 3a, lane 5) which precipitated with a 2D clean-up kit also displayed a sharp band. Still, precipitation with cold acetone did not show clear bands, and the proteins were not effectively extracted with the acetone-phenol (Figure 3a, lane 2) method as no clear bands were detected. Therefore, proteins extracted with the lysis buffer method exhibited reproducible results in bottle gourd leaves. The proteins extracted with the lysis buffer method showed precise band intensities and band strengths (Figure 3b, lane 1), followed by their clean-up. The sucrose and acetone-phenol extractions (Figure 3b, lanes 2 and 5), even after precipitation with acetone and a clean-up kit, did not effectively extract the proteins. When precipitated with ammonium acetate, the extractions with 10% TCA and 0.07% ME + PMSF and TCA with ME (Figure 3b, lanes 3 and 4) showed clear bands in seeds but not in leaves. Due to its reducing nature, SDS extraction (Figure 3b, lane 6) displayed bands but with low intensities. After protein extraction, the supernatant was exposed to different protein precipitation methods, where cold acetone and ammonium acetate-washed proteins showed the smeared bands. With the protein being hydrophobic, acetone reduced the protein’s solubility and amplified its non-polar environment; thus, the proteins coagulated and did not disperse uniformly due to disturbance of the charge-to-mass ratio. Protein precipitated with 2D-clean-up dissolved the pellets entirely and uniformly, and the protein concentration was also enhanced when quantified. 

### 3.4. Polypeptide Composition of Extractable Proteins

In this study, one bottle gourd hybrid HBGH-35 and two parenteral lines, G2 line (M) and Pusa Naveen (F), which differ in their quality and yield traits, were evaluated based on physiological characteristics (Figure 3c–f). SDS-PAGE separated the extractable bottle gourd proteins (leaves and seeds) into multiple components. The electrophoretic protein analysis exhibited a total of 102 protein bands in both parental lines and the hybrid. SDS-PAGE gels resolved 30 µg of protein sample from three biological replicates of the hybrid and two parenteral lines, and electrophoresed proteins were visualized after CBB staining. The banding pattern was consistent across all bottle gourd leaf proteins, resulting in 29 protein bands at various crossing times (see Figure 3c,d for positions of these polypeptides). Some bands were unique and specific markers for genotypes, which permitted their gel electropherograms’ identification and characterization. Six prominent peptide bands were identified with average molecular weights in the range of 39.1–52.4 kDa and 55.0–62.1 kDa (Figure 3c,d). These bands represent the major seed storage proteins and photosynthetic apparatus, accounting for 40–60% of the extracted bottle gourd protein. The 40 kDa polypeptide in hybrid HBGH-35 (Figure 3c) represents the photosynthetic apparatus with higher band strength and band intensity at F4 in comparison to the female line (Figure 3d, lanes 6–10) and the male line (Figure 3d, lanes 1–5). The relative protein expression was more prominent at F4 in all the cultivars, indicating the maximum accumulation of photosynthetic assimilates and storage reserves than at F1, F2, F3, and F5. The significant differences detected in band numbers 4, 37, 38, 42, 49, 62, and 63 differed in appearance and band intensity depending on the storage proteins (Figure 3c,d). Thus, the results indicated that the higher band strengths and apparent band intensities were higher in hybrid HBGH-35, followed by the Pusa Naveen (F) (Figure 3c,d, lanes 6–10) line at the F4 crossing period. 

The proteins extracted from bottle gourd seeds had a different protein pattern than the proteins extracted from the leaves (Figure 3e,f). The gel analysis demonstrated that the protein subunits had a molecular weight ranging from 12.64 to 93.04 kDa. The band intensity and number varied under different crossing periods, hybrid lines, and parental lines. Data showed that hybrid HBG-35 (Figure 3e) was resolved into 20 bands, while the male (Figure 3f, lanes 6–10) and female lines (Figure 3f, lanes 1–5) were resolved into 19 bands, respectively, under different crossing periods. The major protein bands identified in the samples were calculated with a molecular weight of 45.6, 62.8, 85.0, and 87.3 kDa. These bands represent the major seed storage proteins, particularly albumins, prolamins, and gliadins. The 45 kDa polypeptide expressed a significant portion of extractable proteins in bottle gourd seeds. The considerable differences in band numbers 7, 10, 15, 19, and 20 differed in band appearance and intensity. The relative protein expression was more prominent at the F4 crossing period, chiefly in the hybrid (Figure 3e), followed by the female line (Figure 3f, lanes 1–5), indicating the maximum accumulation of photosynthetic assimilates and storage reserves in comparison to F1, F2, F3, and F5. The percentage of each polypeptide varied significantly between bottle gourd lines and crossing periods (F1 to F5). As far as polymorphism is concerned, there were five monomorphic, seven polymorphic, and four unique bands. A detectable band intensity change has been observed in bottle gourd lines under different crossing periods. Thus, hybrid HBGH-35 at the F4 crossing period had a higher band intensity than both parental lines.

### 3.5. Multivariate Analysis of the Datasets

**Figure 4:**
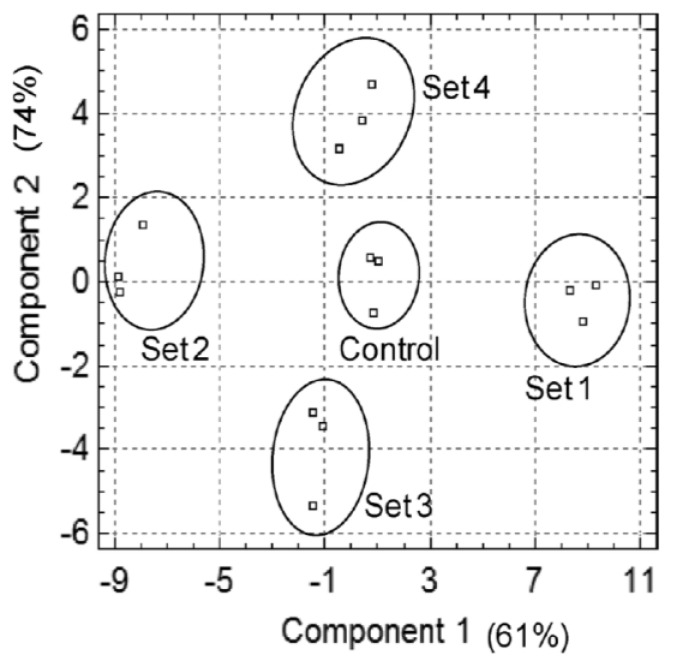
PCA score plot to assess the variance among the protein compositions of the four sets and the control protein mixtures (*n* = 3). Component−1 explains 62.6% of the variability of the data, whereas component 2 is responsible for 13.9% (the total accumulated percentage is 75%).

Multivariate principal component analysis (PCA), performed on all replicates of the four sets (crossing periods F1 to F4) and the control (F0) mixture of proteins, showed five statistically different groups, without overlapping, for sets 1 to 4 and the control (F0) (Figure 4).

### 3.6. Protein Identification and Quantification

In the preliminary experiments, major differences in protein profiles were observed at F4 and F3 crossing periods. Label-free quantification (LFQ) via LC-MS/MS was performed in F4, F3, and F1 (control) crossing periods to detect the variably expressed proteins. Analysis of the LFQ results using MASCOT software revealed more than 100 differentially expressed proteins (DEPs), out of which 20 proteins were upregulated (Table 3) and 12 proteins were downregulated (Table 4), having fold changes of ≥1.5 and ≤0.6, respectively, which were considered for further analysis. The analysis further revealed some important proteins that play a role in seed reserve mobilization related to the source–sink relationship in bottle gourd. Table 3 and Table 4 present a functional analysis of upregulated and downregulated proteins with differential abundance and fold change between genotypes HBGH35 and Pusa Naveen during different crossing periods (F3 and F4) compared to their control (F1). The DEPs were sorted based on their different molecular mechanisms, such as transporters, antioxidative enzymes, photosynthesis, signal transduction, biosynthetic pathways, nucleotide synthesis, carbohydrate metabolism, etc., with a higher protein score, protein coverage, and protein score match (PSM). Different bioinformatic software and tools designed for mass spectrometry-based protein identification and quantification were used to characterize the proteins based on their functions at cellular, biological, and molecular levels using Gene Ontology enrichment, PANTHER 8.0 software, and the KEGG pathway. 

Among the upregulated proteins (Table 3), fold changes in ion transporters, such as H(+)-exporting diphosphatase, HATPase_c domain-containing protein, vacuolar proton pump subunit B, Cation_ATPase_N domain-containing protein, V-type proton ATPase subunit G, and V-ATPase 69 kDa subunit H(+)/Pi co-transporter, at F3 and F4 were more than 2.5 in HBGH35. The proteins for antioxidative enzymes, osmolytes, and molecular chaperones were highly upregulated in SSG 59-3 as compared to control fruits. Other upregulated proteins include metabolic interconversion enzymes, carbon metabolism, photosynthesis, DNA/RNA synthesis, chlorophyll synthesis, and oxidative phosphorylation. Among the downregulated proteins (Table 4), the proteins were from starch and sucrose metabolism, biosynthesis of secondary metabolites, carbon metabolism, gluconeogenesis, and amino acid biosynthesis, with a fold change of ≤0.5. The number of downregulated proteins in Pusa Naveen (♀) was higher compared to HBGH35.

**Figure 5:**
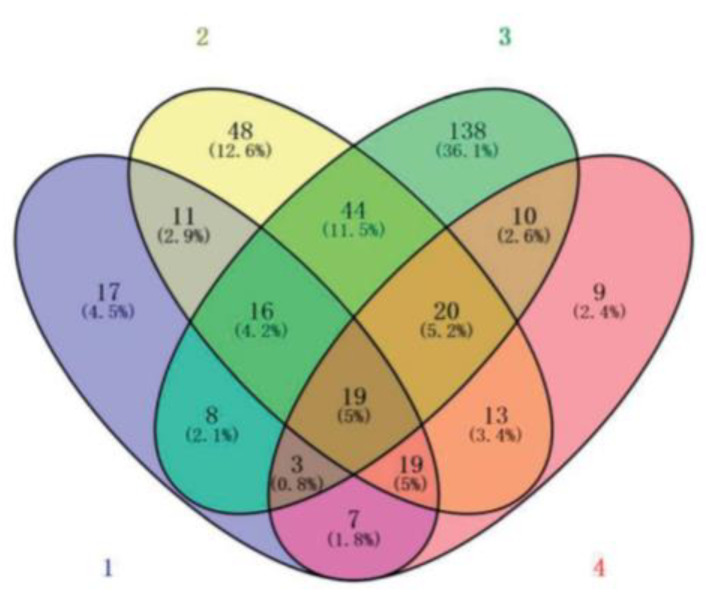
Venn diagram of the number of differentially expressed proteins identified in the bottle gourd proteome under different crossing periods.

Figure 5 compares the changes in differential protein expression under the different crossing periods. The expression of 77 and 13 proteins was significantly reduced and elevated, respectively, following the F1 crossing period (control). The abundance of 141 and 13 proteins was significantly lessened and increased, respectively, following the F4 crossing period, and the abundance of 19 and 13 proteins was significantly lessened and elevated, respectively, following the F3 crossing period.

**Figure 6:**
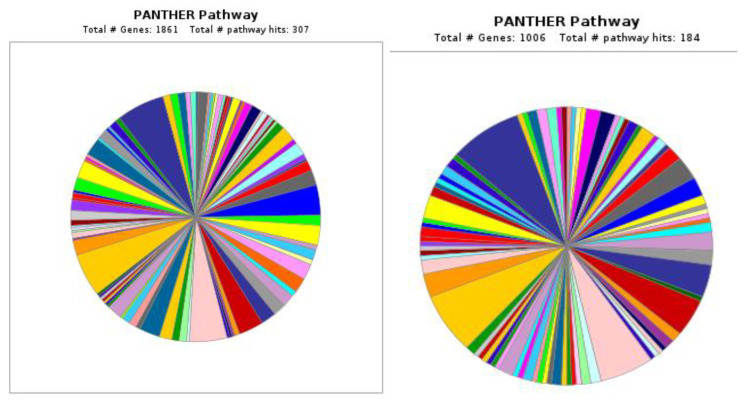
Gene Ontology classification of pathways using PANTHER in HBGH35 and Pusa Naveen (♀).

Using Gene Ontology (GO) online PANTHER (Protein Analysis Through Evolutionary Relationships) 8.0 software, a total of 1861 pathway hits in HBGH-35 and 1006 pathway hits in Pusa Naveen were observed (Figure 6). PANTHER is an approach to outlining the genes and gene product properties that are shared across species. The PANTHER classification system (http://pantherdb.org/ 15 October 2022) has been designed to classify proteins (and their corresponding genes) to facilitate high-throughput analysis.

**Figure 7:**
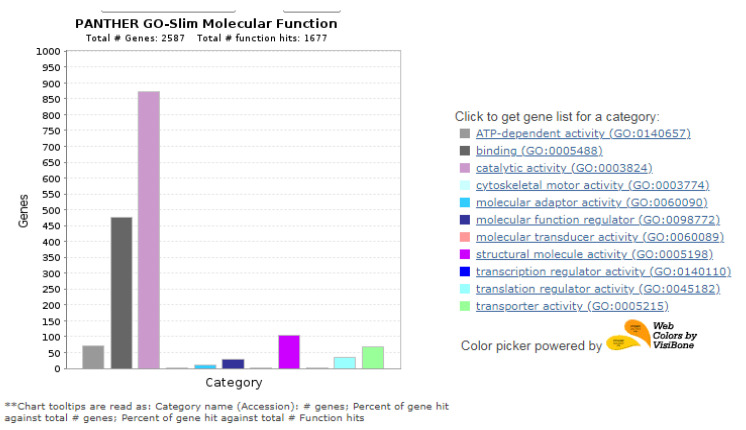
Gene Ontology classification of proteins in HBGH35 and Pusa Naveen (♀) based on their molecular function.

**Figure 8:**
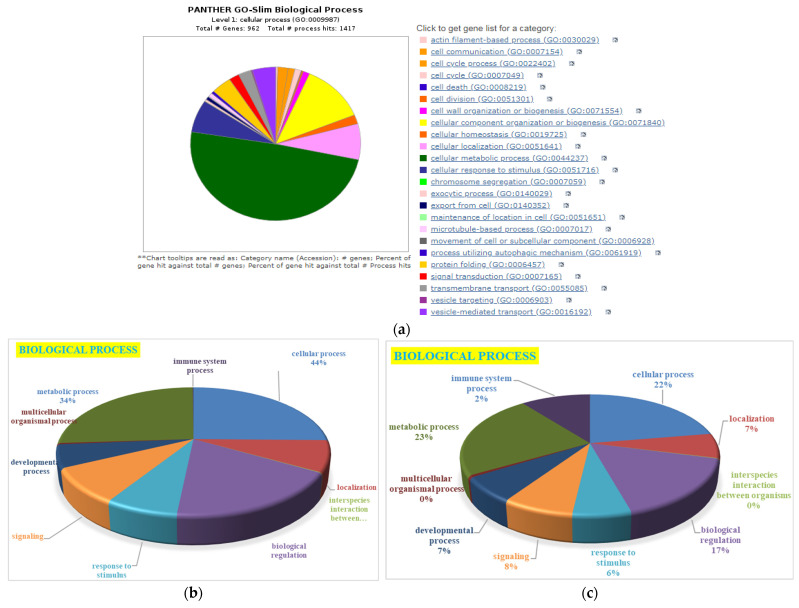
Gene Ontology classification of proteins with: (**a**) process hits, (**b**) HBGH35, and (**c**) Pusa Naveen (♀) based on their biological processes.

**Figure 9:**
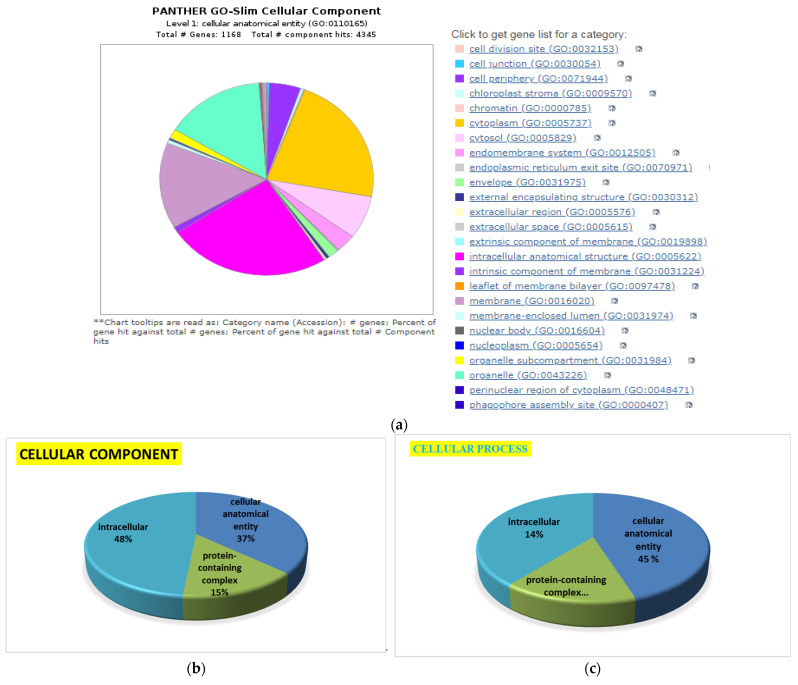
Gene Ontology classification of proteins: (**a**) process hits, (**b**) HBGH35, and (**c**) Pusa Naveen (♀) based on their cellular level.

**Figure 10:**
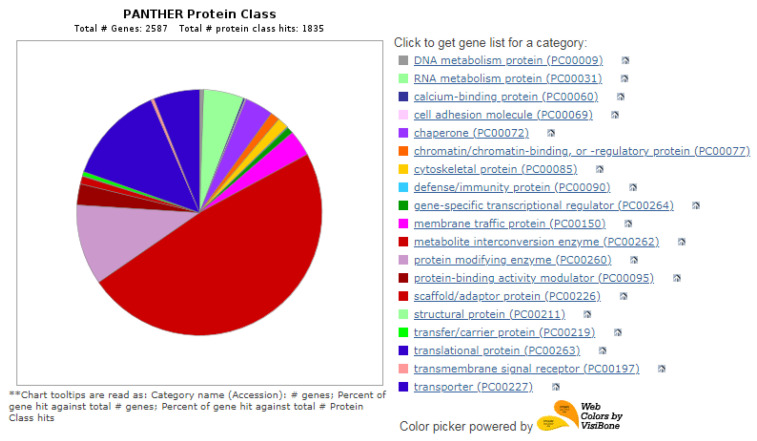
Gene Ontology classification of proteins in HBGH35 and Pusa Naveen (♀) based on their molecular function and metabolic interconversion enzymes.

**Figure 11:**
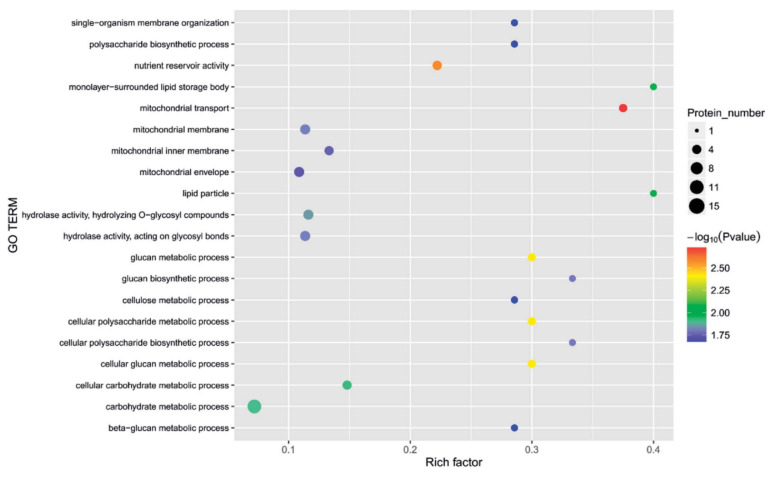
The functions of the main differential proteins in different crossing periods.

**Figure 12:**
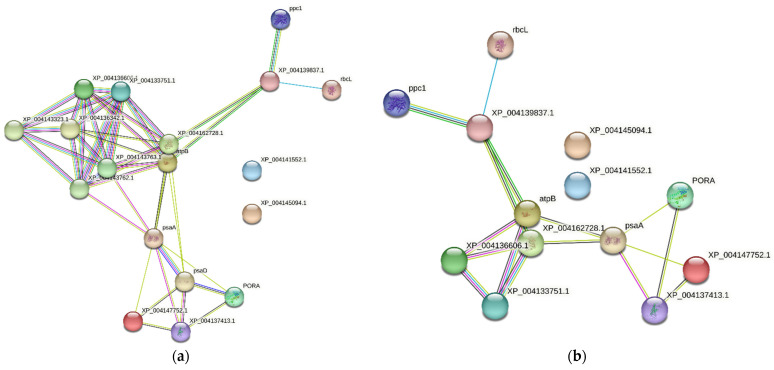

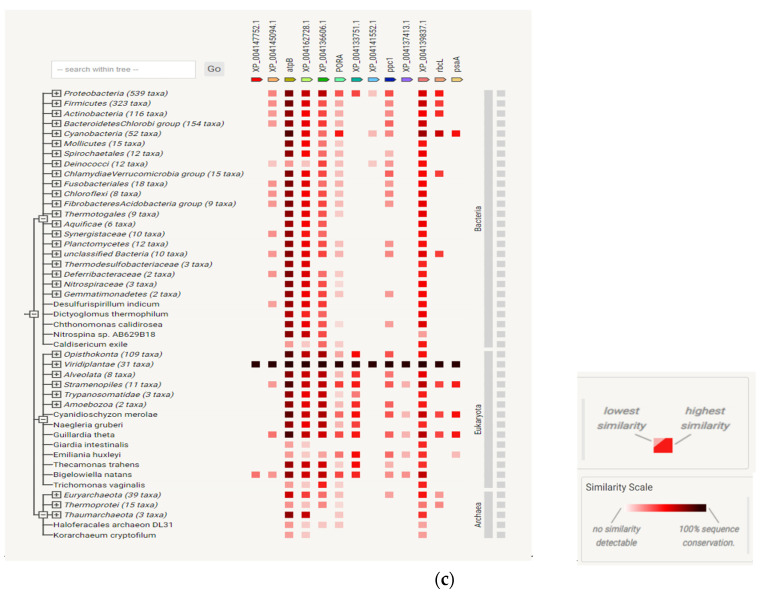
STRING analysis of (**a**) more abundant, (**b**) less abundant, and (**c**) gene co-occurrence patterns across genomes shows similarities.

The functional characterization of identified proteins was based on Gene Ontology (GO) using the PANTHER 8.0 platform, which generated information regarding cellular localization and metabolic and biological processes. The classification of proteins was based on cellular components, molecular function, and biological processes (Figure 7, Figure 8, Figure 9, Figure 10 and Figure 11), and to cluster them, we exploited the K-means clustering approach of the web tool STRING (https://string-db.org/ 17 October 2023). The obtained results are shown in Figure 12a (more abundant proteins) and Figure 12b (less abundant proteins), and Figure 12c shows gene co-occurrence across genomes. Gene ontology protein classification in HBGH35 and Pusa Naveen (♀) classify DEPs based on their molecular function (Figure 7). Around 1677 molecular function hits were observed across 2587 genes. About 62% of the proteins were involved in a catalytic role (GO: 0003824), which includes antioxidative enzymes, molecular chaperons, osmoprotectants, sugars, antioxidants, etc., and 25% of the proteins belonged to the binding protein class (GO: 0005488), which includes proteins that regulate abiotic stress conditions, signal transduction pathways, and post-transcriptional processes such as splicing regulation, mRNA transport, and mRNA translation modulation. Other proteins involved in molecular function include those involved in molecular adaptor activity (GO: 0060090), molecular transducer activity (GO: 0060089), structural molecule activity (GO: 0005198), and transporter activity (GO: 0005215). After identifying the molecular functions of the DEPs, further analysis was performed to determine the protein classes for the proteins involved in molecular function.

Around 1417 hits to biological processes were observed from 962 genes (Figure 8a) based on biological processes (Figure 8b) in HBGH-35, and the proteins were classified into 12 categories: those involved in cellular processes (44%), metabolic processes (35%), biological regulation (7%), localization (7%), response to stimulus (6%), biological phase (1.8%), reproductive processes (1.2%), signaling (1%), developmental processes (0.6%), reproduction (0.5%), multicellular organismal processes (0.4%), and immune system processes (0.4%) (Figure 8a). Majority of the proteins in Pusa Naveen (♀) were involved in the metabolic processes (23%), cellular processes (22%), biological regulation (17%), signaling (8%), response to stimulus (6%), localization (7%), developmental processes (7%), immune system response (2%), and multicellular organismal processes (0.1%) (Figure 8c).

The cellular component classification in HBGH35 and Pusa Naveen (♀) (Figure 9) revealed that in HBGH35, majority of proteins (42.5%) were found in the intracellular region, followed by cellular and anatomical activity (44.7%) and protein-containing complex (13.7%), while in Pusa Naveen (♀), four categories were observed: intracellular (43%), biosynthetic process (23%), cellular and anatomical activity (21%), and protein-containing complex (11%). Salinity under osmotic stress, salinity-induced cellular protein conversion to involvement in intracellular responses, which involved the metabolic and several signal transduction pathways, was followed by a cascade of intracellular signals via binding proteins and protein complex networks.

Among the metabolic interconversion enzymes (PC00262), Gene Ontology classification further classified 1835 proteins (Figure 10). Protein-modifying enzymes (PC00260) are the first category of metabolic interconversion enzymes, followed by transporters (PC00227) and translational proteins (PC00263). The oxidoreductase consists of alcohol dehydrogenase, catalase, choline oxidase, and enzymes involved in glycolysis, the TCA cycle, oxidative phosphorylation, and amino acid metabolism. The transferases include glutathione reductase, glutathione, glutathione S-transferases, and coenzyme A (CoA). Other categories of enzymes included were calcium-binding proteins (PC00060), chaperones (PC00072), membrane traffic proteins (PC00150), chromatin binding/regulatory proteins (PC00077), and RNA metabolism proteins (PC00031). The overall result of protein identification and the results of differential proteins are shown in Figure 11.

## 4. Discussion

Due to climate change in the last decade, food production has been adversely affected worldwide and has become a significant threat to food security. Finding an appropriate crossing period that preserves high seed quality and yield may enhance high-quality seed production under different environmental conditions. It is indispensable because suitable crossing selection is essential for effective seed production with robust-quality seeds. Seed vigor determines the degree of aliveness and is depicted by the complicated interplay between genetic and environmental factors [30]. Bottle gourd grows well in areas with a rainfall range of 400–1500 mm per annum; however, moderate rather than excessive soil water is desired for a good harvest [31]. Therefore, bottle gourd is intolerant of waterlogging. Our results are well-supported by Grubben and Denton [32], who reported that bottle gourd grows well at warm temperatures (25–35 °C). The optimum germination temperature is between 20 and 25 °C. Flowering is highly sensitive to the photoperiod. Short days coupled with low night temperatures and high relative humidity promote male flowers’ development, while the reverse promotes female flowers [31]. Not much information is available on the production of bottle gourd. Agronomic practices and physiological attributes that encourage more female flower production than male flower production might increase yields. Thus, our results might help to determine the optimal growth conditions to maximize fruit and seed sets.

At a temperature of 34 °C and a relative humidity of 87%, the F4 crossing period had maximum seed potential. The interaction plot between the bottle gourd parental lines and the hybrid revealed that both hybrid HBGH-35 and F4 crossing periods had maximum seed vigor I and seed vigor II. Our results are well-supported by several other researchers [33,34].

With the protein being hydrophobic, acetone reduced the protein’s solubility and amplified its non-polar environment; thus, the proteins coagulated and did not disperse uniformly due to disturbance of the charge-to-mass ratio [7]. Protein precipitated with 2D-clean-up entirely and uniformly dissolved the pellets, and the protein concentration was also enhanced when quantified. The variations among extractable proteins were also reported by several authors [35,36,37], but no reports are available for comparative proteome analysis in bottle gourd. Henceforth, our results demonstrated that protein extraction using lysis buffer followed by its clean-up was the most efficient and reliable protocol for extracting protein from bottle gourd (leaves and seeds). Thus, standardization of extraction methods in bottle gourd holds potential in future understanding of the seed development process via identification of novel proteins involved in seed set.

The banding pattern was similar among all bottle gourd leaf proteins for a total of ≤29 protein bands at different crossing periods. Some bands were unique and specific markers for genotypes, which permitted their gel electropherograms’ identification and characterization. Other researchers have also reported the genotypic variation among the extracted proteins based on their polypeptide composition [38].

Protein analysis using SDS-PAGE revealed a positive correlation between seed storage protein accumulation and their differential expression during different crossing periods in the current study. Furthermore, Capouchová et al. [38] used SDS-PAGE to investigate the proteome behavior in oat and sorghum genotypes. The authors reported that the higher proportion of proteins was characterized by prolamins and glutelins, approximately 75.02% and 50.63% of total seed storage proteins, respectively. This study is also supported by several other authors [36,39]. Srivastava et al. [40] and Punia et al. [7] conducted a molecular diversity pattern in bottle gourd using a RAPD marker and SDS-PAGE. Results showed that a total of 60 bands were observed, of which 60.29% were polymorphic and had a similarity coefficient of 0.68 on a similarity matrix. Our study may act as a baseline for detecting and identifying proteins under different crossing periods for quality seed production and might be exploited in molecular future breeding programs in developing bottle gourd cultivars with high yield and good kernel quality, with full vigor potential. Future insights about direct regulatory networks in bottle gourd would require comprehensive functional analysis of such proteins.

Proteomic studies have emerged as a new platform to unravel important relationships between protein abundance and plant stress acclimatization, allowing fast discovery and precise protein profiling under different abiotic stresses [2,41,42]. The leaves play a significant role in transporting water and minerals from the roots to the aerial parts. The electrophoretic profiling of proteins via SDS-PAGE provides a preliminary foundation for protein patterning under abiotic stress conditions. Label-free quantitative (LFQ) proteomics has allowed rapid identification, expression dynamics, and post-translational modifications (PTMs) of proteins [43]. Several reports on comparative proteomics have been explored in sorghum to study the abiotic stress responses and identify the prominent protein groups and protein classes as being salinity- or drought-responsive via several bioinformatics tools, such as Gene Ontology (GO), PANTHER, KEGG pathway, etc. [35,44,45]. Bandehagh et al. [46] studied salt-responsive proteins in canola leaves using a proteomic technique. The differentially expressed proteins involved various processes, including oxidative stress, energy production, electron transport signal transduction, translation, phosphate metabolic processes, and photosynthesis [47,48,49]. However, majority of these reports so far have relied on conventional protein extraction approaches [48] and gel-based protein abundance studies, including two-dimensional difference gel electrophoresis (2D-DIGE) or two-dimensional gel electrophoresis (2DE), followed by a matrix-assisted laser desorption/ionization-time of flight mass spectrometry (MALDI-TOF-MS) approach for individual protein identification. Nevertheless, consistent and reproducible results from 2DE or 2D-DIGE were challenging, and poor resolution of integral membrane proteins is another concern [23,50,51]. As a result, gel-free approaches in plant proteomics are now being used [52], which include liquid chromatography (LC) and ion-exchange chromatography, followed by de novo sequencing of the peptide fragments by MS/MS, and ultimately offer high-throughput analysis of the proteome profile, providing a snapshot of the major protein constituents.

## 5. Conclusions

The quantitative proteomics analysis of bottle gourd parental lines under different crossing periods revealed the mechanism of source–sink strength for superior seed quality. The standardization and comparative analysis of different protein extraction methods provided reproducible and high-quality SDS-PAGE results in both leaves and seeds at the F4 crossing period. The seed vigor followed a similar pattern as the differential protein expression by SDS-PAGE. Analysis of the LFQ results using MASCOT software revealed more than 100 differentially expressed proteins (DEPs), of which 20 proteins were upregulated and 12 proteins were downregulated, with fold changes of ≥1.5 and ≤0.6, respectively. The analysis further revealed some important proteins that play a role in seed reserve mobilization related to the source–sink relationship in bottle gourd. The DEPs were sorted based on their different molecular mechanisms, such as transporters, mineral deposition, antioxidative enzymes, photosynthesis, signal transduction, biosynthetic pathways, nucleotide synthesis, carbohydrate metabolism, etc., with a higher protein score, protein coverage, and protein score match (PSM). Future research aims to further understand the dynamic molecular interactions of protein candidates, especially those with specific bottle gourd seed attributes, and the modalities that enable this crop to be a valuable medicinal and horticultural crop with high nutritional value.

## Figures and Tables

**Table 1:** Weather data during the field experiment.

Month	Maximum	Minimum	RH	RH	BRI	PAN	RAIN	Average
	Temperature	Temperature	(%)	(%)	SUN	Evaporation	Fall	WS
	°C	°C	M	E	HRS	(mm)	(mm)	KM/H
July	34.4	27.0	90.7	70.9	6.5	4.6	2.6	7.3
August	34.7	26.3	89.7	69.3	6.3	4.2	3.1	5.6
September	34.9	23.5	87.2	49.5	6.8	4.2	1.9	2.9
October	35.0	17.2	84.8	28.0	6.6	3.6	0.0	1.9
November	27.2	10.8	90.1	39.8	3.4	2.8	0.0	2.0
December	23.8	7.3	89.7	34.5	3.7	1.4	0.0	0.7
Mean	31.7	18.7	88.7	48.7	5.5	3.5	1.3	3.4

BRI = bright sunshine hours; PAN = evapotranspiration.

**Table 2:** Temperature conditions under different crossing periods of bottle gourd during the field experiment.

	DOC	Max. T (°C)	Min. T (°C)		DOC	Max. T (°C)	Min. T (°C)
F_1_	4-September-2017	35.8	25.6	F_2_	11-September-2017	37.2	26.5
	5-September-2017	34.9	24		12-September-2017	34.9	24.5
	6-September-2017	34.6	25.3		13-September-2017	35.9	25.6
	7-September-2017	35.9	22.5		14-September-2017	36.4	26
	8-September-2017	33.4	24.5		15-September-2017	35.4	23.9
	9-September-2017	34.2	25		16-September-2017	35.8	23.9
	10-September-2017	34.4	25.9		17-September-2017	32.0	24.2
	Mean	34.7	24.7		Mean	35.4	24.9
F_3_	18-September-2017	35.6	20	F_4_	25-September-2017	34.4	22.7
	19-September-2017	36.6	19.9		26-September-2017	36.4	23.5
	20-September-2017	36.8	19		27-September-2017	36	22.9
	21-September-2017	36.8	22.9		28-September-2017	36.9	22.9
	22-September-2017	36.4	24.3		29-September-2017	36.6	20.5
	23-September-2017	33.4	24		30-September-2017	37	19.8
	24-September-2017	30.6	20.2		1-October-2017	37.4	18.5
	Mean	35.2	21.5		Mean	36.4	21.5
F_5_	3-October-2017	37	18.8				
	4-October-2017	36.4	19.5				
	5-October-2017	36.9	19				
	6-October-2017	36.2	19.5				
	7-October-2017	36.4	18.2				
	8-October-2017	35.6	18				
	Mean	36.5	19.0				

**Table 3:** LFQ (MASCOT) list of upregulated proteins in HBGH-35 and Pusa Naveen (♀) at various crossing times.

S. No.	Accession	Protein Description	MW (kDa)	pI	Protein score	SC (%)	Peptides	PSM	Fold Change						KEGG Pathway
									HBGH35			Pusa Naveen (♀)			
									F1	F3	F4	F1	F3	F4	
	Ion Transporters														
1.	A0A194YHK2	H(+)-exporting diphosphatase	79.7	5.33	467.22	13	3	168	1.45	4.83	6.15	1.21	2.44	2.65	Plant–pathogen interaction; protein processing in the endoplasmic reticulum
2.	C5YBL4	HATPase_c domain-containing protein	81.7	5.11	197.27	9	3	115	1.62	2.66	4.11	0.27	0.64	1.24	Phagosome; metabolic pathways; oxidative phosphorylation
3.	C5WP97	Cation_ATPase_N domain-containing protein	115.4	5.58	246.11	10	10	92	1.21	3.48	5.2	0.19	0.23	0.13	Phagosome; metabolic pathways; oxidative phosphorylation
4.	A0A194YNQ1	Vacuolar proton pump subunit B	54.2	5.36	998.47	17	17	392	1.2	3.42	3.05	0.28	0.84	1.02	Phagosome; metabolic pathways
5.	A0A1B6Q818	Glutathione transferase	24	6.52	582.35	7	7	193	1.34	4.26	3.55	1.02	0.51	0.36	Phagosome; oxidative phosphorylation; metabolic pathways
6.	C5YF66	V-type proton ATPase subunit G	12.3	6.13	18.57	2	2	7	2.67	3.46	4.9	1.64	2.64	2.32	Phagosome; metabolic pathways; oxidative phosphorylation
7.	C5YX05	V-type proton ATPase subunit C	42.7	5.78	446.02	13	6	170	2.13	5.94	6.54	1.24	3.91	4.09	Phagosome; metabolic pathways
	Antioxidative enzymes, osmolytes, chaperons														
8.	A0A1B6PMT8	PEROXIDASE_4 domain-containing protein	38.3	8.19	372.99	11	11	155	1.23	3.23	4.35	1.2	2.1	2.8	Glutathione metabolism
9.	C5X6H6	L-ascorbate peroxidase	27.1	5.36	863.16	9	8	318	1.2	7.37	4.32	0.32	0.54	0.72	Arachidonic acid metabolism; glutathione metabolism
10	Q6JAG4	Glutathione peroxidase	18.4	7.08	326.46	7	6	122	1.17	3.32	5.11	1.2	1.08	2.34	Phagosome; metabolic pathways
11	C5WWX0	Glutathione reductase	59.3	7.56	458.25	14	14	209	2.04	4.23	6.23	1.17	2.43	3.01	Glutathione metabolism
12	A0A1B6QQQ9	Catalase	56.6	7.28	656.35	15	12	268	1.19	4.3	6.5	2.31	3.92	4.09	Scavenging
13	A0A1B6Q707	Delta-1-pyrroline-5-carboxylate synthase	78.3	6.42	193.73	11	10	83	1.04	6.64	4.8	1.24	2.27	3.01	Arginine and proline metabolism; biosynthesis of secondary metabolites; metabolic pathways
14	C5WSJ9	Proline dehydrogenase	53	7.69	2.48	1	1	1	4.64	6.54	6.64	1.07	2.64	3.64	Osmoregulation
15	C5Y3J4	LEA_2 domain-containing protein	24	9.11	42.13	2	2	20	1.35	3.77	4.51	1.03	2.62	4.43	Osmoregulation; molecular chaperons
16	A1E9W3	30S ribosomal protein S3, chloroplastic	25.9	9.74	102.5	5	5	51	1.2	3.2	4.6	1.3	2.2	3.3	Ribosome
17	A0A1B6PDG7	Diaminopimelate epimerase	37.8	6.54	106.48	6	6	46	2.11	4.29	5.22	2.08	3.01	2.32	Cysteine and methionine metabolism; metabolic pathways; biosynthesis of amino acids; sulfur metabolism; biosynthesis of secondary metabolites; carbon metabolism
18	A0A1B6P8M2	Pyruvate, phosphate dikinase	102.4	5.73	3300.97	44	44	1234	2.12	4.6	5.13	1.26	2.43	3.0	Protein export
19	C5YUG2	Starch synthase, chloroplastic/amyloplastic	103	6.49	101.91	6	6	32	2.39	2.75	3.05	1.14	3.04	4.2	Biosynthesis; carbohydrate metabolism
20	C5YD77	Glucose-6-phosphate 1-dehydrogenase	66.6	8.35	107.16	5	3	50	1.14	2.6	3.16	1.26	2.26	3.46	Glucose metabolism

**Table 4:** LFQ (MASCOT) list of downregulated proteins in HBGH35 and Pusa Naveen (♀) under different crossing periods.

S. No.	Accession	Protein Description	MW (kDa)	pI	Protein Score	SC (%)	Peptides	PSM	Fold Change						KEGG Pathway
									HBGH35			Pusa Naveen (♀)			
									F1	F3	F4	F1	F3	F4	
1.	A0A1Z5RLT7	Ribosomal_L16 domain-containing protein	20.8	10.18	192.37	6	3	82	−0.74	−0.72	−0.71	−0.81	−2.92	−0.01	Ribosome
2.	A1E9W3	30S ribosomal protein S3, chloroplastic	25.9	9.74	102.5	5	5	51	−0.1	−0.34	−0.11	-	-	-	Ribosome
3.	C5WYH8	Glutamyl-tRNA synthetase	81.2	7.44	317.85	11	4	133	−0.31	−0.2	−0.47	−0.51	−0.91	−0.61	Metabolic pathways; biosynthesis of secondary metabolites; aminoacyl-tRNA biosynthesis; porphyrin and chlorophyll metabolism
4.	C5XFP1	Cysteine synthase	42.1	8.28	481.2	11	11	170	-	-	-	−0.33	0	−0.44	Cysteine and methionine metabolism; metabolic pathways; biosynthesis of amino acids; sulfur metabolism; biosynthesis of secondary metabolites; carbon metabolism
5.	C5XDW6	UDP-arabinopyranose mutase	40.8	7.05	212.84	7	2	103	-	-	-	−0.43	−0.14	−0.27	Amino sugar and nucleotide sugar metabolism
6.	C5YAY1	Protein kinase domain-containing protein	67.4	7.44	69.27	3	3	27	-	-	-	−0.35	−0.35	−0.16	Signal transduction
7.	C5Y2Z7	E1 ubiquitin-activating enzyme	116.7	5.36	629.61	22	2	258	-	-	-	−0.99	−0.17	−0.32	Ubiquitin-mediated proteolysis
8.	C5YDP0	40S ribosomal protein S8	24.9	10.39	390.36	6	2	155	-	-	-	−0.02	−0.91	−1	Ribosome
9.	C5XZJ2	Vacuolar protein sorting-associated protein 29	20.9	6.6	4.8	1	1	2	-	-	-	−6.64	−6.64	−0.3	Endocytosis
10.	C5WZ11	Glutathione transferase	25.7	7.56	95.7	3	1	36	-	-	-	−1.67	−6.64	−0.11	Glutathione metabolism
11.	C5XBD4	Phosphopyruvate hydratase	50.5	6.29	168.11	6	6	66	−0.05	−0.01	−0.16	−0.05	−1.57	−0.57	RNA degradation; carbon metabolism; glycolysis/Gluconeogenesis; biosynthesis of amino acids; metabolic pathways; biosynthesis of secondary metabolites
12.	C5Y9W4	14_3_3 domain-containing protein	29.6	4.81	959.55	14	9	347	−0.22	−0.72	−0.3	0-.32	−0.03	0.19	

## Data Availability

Not applicable.
